# The Impact of Green Physical Crosslinking Methods on the Development of Sericin-Based Biohydrogels for Wound Healing

**DOI:** 10.3390/biomimetics9080497

**Published:** 2024-08-16

**Authors:** Maria C. Arango, Natalia Jaramillo-Quiceno, José David Badia, Amparo Cháfer, Josep Pasqual Cerisuelo, Catalina Álvarez-López

**Affiliations:** 1 Agroindustrial Research Group, Department of Chemical Engineering, Universidad Pontificia Bolivariana, Cq. 1 #70-01, Medellín 050031, Colombia; 2 Materials Technology and Sustainability (MATS), Department of Chemical Engineering, Universitat de València, Av. de la Universitat s/n, 46100 Burjassot, Spain

**Keywords:** hydrogel, silk sericin, poly (vinyl alcohol), green crosslinking, annealing treatment, ethanol vapor, water vapor annealing

## Abstract

Silk sericin (SS)–based hydrogels show promise for wound healing due to their biocompatibility, moisture regulation, and cell proliferation properties. However, there is still a need to develop green crosslinking methods to obtain non-toxic, absorbent, and mechanically strong SS hydrogels. This study investigated the effects of three green crosslinking methods, annealing treatment (T), exposure to an absolute ethanol vapor atmosphere (V.E), and water vapor (V.A), on the physicochemical and mechanical properties of SS and poly (vinyl alcohol) (PVA) biohydrogels. X-ray diffraction and Fourier-transform infrared spectroscopy were used to determine chemical structures. Thermal properties and morphological changes were studied through thermogravimetric analysis and scanning electron microscopy, respectively. The water absorption capacity, mass loss, sericin release in phosphate-buffered saline (PBS), and compressive strength were also evaluated. The results showed that physical crosslinking methods induced different structural transitions in the biohydrogels, impacting their mechanical properties. In particular, V.A hydrogen presented the highest compressive strength at 80% deformation owing to its compact and porous structure with crystallization and bonding sites. Moreover, both the V.A and T hydrogels exhibited improved absorption capacity, stability, and slow SS release in PBS. These results demonstrate the potential of green physical crosslinking techniques for producing SS/PVA biomaterials for wound healing applications.

## 1. Introduction

Hydrogels are three-dimensional polymer networks that can absorb thousands of times their dry weight in water [1]. The hydrophilic groups or segments of the polymer network of these materials have a high affinity for water even under neutral conditions, and both their stability and flexibility are due to the reticulated structure and intertwining of the polymer chains that constitute them [2]. Due to the good properties of hydrogels such as high-water retention, controllable physicochemical properties, and high elasticity, they have been used as biomaterials in various biomedical applications, especially in wound healing, to improve exudate absorption and maintain a favorable environment during the healing process [1,3]. Most commercial hydrogel-type biomaterials used for wound healing are based on synthetic polymers [4,5]; therefore, they exhibit low adhesion and cell proliferation. Thus, natural polymers have been investigated to increase the biocompatibility of hydrogels used for wound healing [6,7]. One large family of natural polymers are polysaccharides, such as alginate and chitosan, which are abundant and exhibit a wide spectrum of biological effects. Alginate (ALG) is a non-immunogenic polyanion, and polymeric formulations based on it have been investigated as wound dressings, proving to accelerate the healing mechanism compared with traditional dressings [8]. Chitosan (CHI) is a cationic polymer widely used in the biomedical industry because of its low toxicity and antibacterial properties [9]. Many polysaccharides are highly hydrophilic molecules with low stability and mechanical strength. Hence, different authors have implemented chemical crosslinking methods, such as the combination of 1-ethyl-3-(-3-dimethylaminopropyl) carbodiimide (EDC) and N-hydroxysuccinimide (NHS), to enhance the structural and mechanical stability of biomaterials [10].

Likewise, protein-based hydrogels are excellent candidates for biomedical applications because their properties are similar to those of the extracellular matrix [11]. Silk sericin (SS), a protein easily isolated from silk cocoons, can be used in the development of biomaterials for skin regeneration, since it has important properties, such as antioxidant capacity, moisture regulating capacity, biocompatibility, biodegradability, and low cost compared to traditional materials, and it can favor both cell adhesion and cell proliferation. Importantly, SS has certain intrinsic antimicrobial properties, which may be due to its cationic nature, the presence of cysteine with a sulfhydryl group in its composition, and other small components [12,13]. Thus, antimicrobial SS composite biomaterials have been developed. However, dry biomaterials produced by SS are brittle and rigid, and thus their mechanical properties should be improved to allow the development of adequate biomaterials.

Because SS has polar side chains with different functional properties, such as amine, hydroxyl, and carboxy groups, their presence can foster chemical interactions with other polymers through blending and crosslinking methods [14,15], thereby improving the mechanical resistance of sericin-based biomaterials. Previous studies have successfully developed hydrogels by blending SS with polymers, such as carboxymethyl cellulose, gelatin, polyacrylamide, or poly (vinyl alcohol) (PVA) [16,17,18]. PVA, a synthetic polymer, has received significant attention for biomedical applications because of its notable properties, such as biocompatibility, chemical stability, affordability, and the ability to form hydrogels with high moisture retention [19]. This polymer is frequently mixed with natural polymers to increase their mechanical performance [2,20,21,22]. An SS/PVA blend has been the most outstanding because of their hydrogen bonding interactions, where PVA provides mechanical stability, while SS preserves the swelling capacity and, in some cases, can be released to promote wound healing [17,23,24]. In addition, physical and chemical modifications have been made to further improve limitations such as the water stability of biomaterials [18,25]. Chemical crosslinking is the most common and is expected to provide desired properties [26]. However, several crosslinking agents used to improve the properties of hydrogels have been reported to be cytotoxic and provide limited improvements [27,28,29]. On the other hand, physical crosslinking techniques do not require the use of chemical agents, thus avoiding toxicity problems commonly associated with this type of compound [30]. Physical crosslinking techniques, such as solvent vapor (with ethanol or water) and annealing treatment, have been evaluated in biomaterials based on fibroin, the other protein present in silk cocoons, or in scaffolds only made by sericin [31,32,33]. Although different green crosslinking techniques have been studied, there has not been a study that compares different methods in the formation of the same material.

Therefore, this study aimed to evaluate the effect of three physical crosslinking methods on the development of SS/PVA-based hydrogels using annealing treatment (T), exposure to an absolute ethanol vapor atmosphere (V.E), and water vapor annealing (V.A). Non-crosslinked hydrogels were also investigated and used as a control. The obtained hydrogels were characterized by X-ray diffraction and Fourier-transform infrared spectroscopy (FTIR ATR) to reveal their chemical structure. Thermal properties and morphological changes in the crosslinked and non-crosslinked hydrogels were studied by thermogravimetric analysis (TGA) and scanning electron microscopy (SEM), respectively. In addition, the water absorption capacity and mass loss in phosphate-buffered saline (PBS) were evaluated at 24 h. The sericin release from hydrogels in PBS was also evaluated at 24 and 48 h. Finally, the compressive strength of the hydrogels was determined by a compression test.

## 2. Experimental Section

### 2.1. Materials

Silkworm cocoons, provided by the Corporation for the Development of the Cauca Sericulture (CORSEDA) in Colombia, were cut into small pieces after removing the dry pupa and some impurities. The PVA used in this study was a commercial product purchased from Sigma-Aldrich (St. Louis, MO, USA) with a degree of hydrolysis higher than 98–99% and a molecular weight of 146–186 kDa. The biuret reagent was acquired from the laboratory of the Bolivarian Pontifical University (Medellín, Colombia) and used for the quantification of proteins in solution.

### 2.2. Preparation of Sericin Powder

SS was extracted from previously chopped cocoons using a high-temperature and pressure-degumming technique. Briefly, small pieces of silkworm cocoons were put into distilled water within a bath ratio of 1:30 (g/mL) and then autoclaved in an AV model autoclave (Phoenix Ltda., Araraquara, Brazil) at 120 °C for 30 min. The obtained SS extract was filtered using a fabric mesh to discard the remaining cocoon pieces and remove the particulate material and possible impurities present in the solution. Finally, a spray drying process was carried out using a BUCHI brand equipment (B-290 BÜCHI Labortechnik AG, Flawil, Switzerland), maintaining an inlet temperature of 160 °C, an airflow of 40 m^3^/h, and a flow rate of sericin solution of 6.3 mL/min. The sericin extract powder obtained was stored in a desiccator until use.

### 2.3. Preparation of SS/PVA Hydrogels

Sericin powder was dissolved in distilled water to obtain a 2% (*w*/*v*) sericin solution according to the results obtained in previous studies [33]. The sample was dissolved in an autoclave under the same conditions as described above for the extraction process. PVA powder was dissolved in distilled water to a final concentration of 2% (*w*/*v*) under constant stirring at 85 °C until complete dissolution. The PVA solution was added to the sericin solution at the same temperature with continuous magnetic stirring (IKA Works Inc., Wilmington, NC, USA) for approximately 20 min to promote the blending of sericin and PVA. The solution was mixed in a 50:50 SS/PVA (*v*/*v*) ratio, and samples of 100% SS and 100% PVA were used as controls. The solutions obtained were poured into plates with 1.5 mL wells, which were frozen at −80 °C for 24 h. Subsequently, frozen samples were lyophilized (Labconco Corporation, Kansas, KS, USA) for 48 h. The obtained samples were stored in a desiccator for subsequent characterization.

### 2.4. Crosslinking of SS/PVA Hydrogels

**Figure 1:**
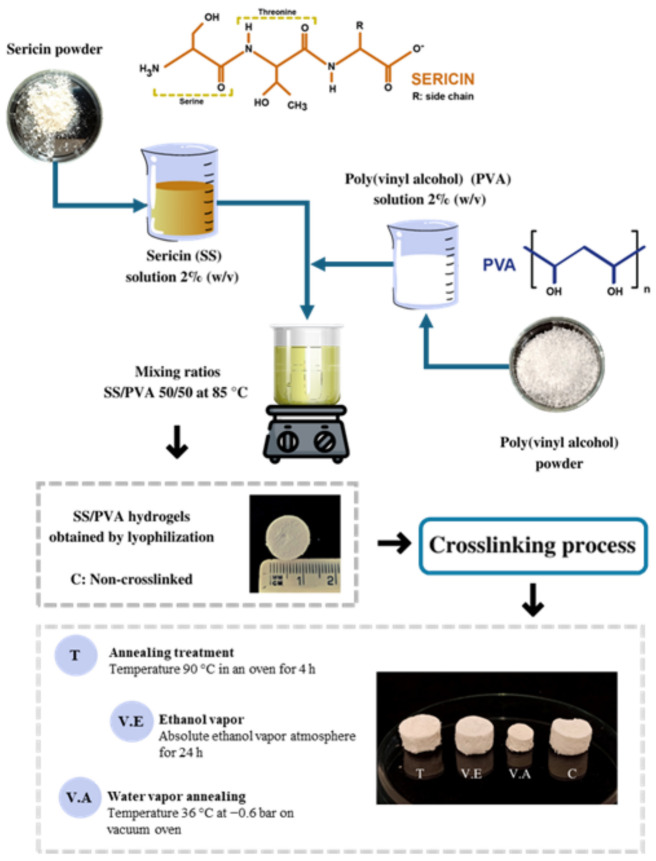
An illustration of the development of crosslinked (T, V.E, and V.A) and non-crosslinked (C) SS/PVA biohydrogels.

Freestanding SS/PVA hydrogels were crosslinked using three different methods: annealing treatment, ethanol vapor, and water vapor annealing. For annealing treatment, the samples were annealed at 90 °C in an oven for 4 h (BOV-V30F, Biobase, Jinan, China) [24,34,35,36]; for ethanol vapor, the samples were exposed to an absolute ethanol vapor atmosphere for 24 h at 25 °C [37,38,39,40,41]. For water vapor annealing, the temperature controller of the vacuum oven (Vaciotem-T, Selecta, Barcelona, Spain) was first set at the desired annealing temperature (36 °C). Subsequently, the samples were transferred, and a vacuum was applied until homogeneous water vapor was obtained (−0.6 bar) [31,42]. Treatment was performed for 24 h. All of these conditions were selected based on preliminary tests and reports by other authors [31]. Non-crosslinked hydrogels were also investigated and used as control. The samples were labelled as follows: T, annealing treatment; V.E, ethanol vapor; V.A, water vapor annealing; and C: control non-crosslinked SS/PVA hydrogels. Figure 1 illustrates the process and the obtained hydrogels.

### 2.5. Characterization Methods

The characterization tests performed on the samples are described below.

#### 2.5.1. X-ray Diffraction

X-ray diffraction (XRD) curves were obtained using a Cu source with a diffraction range (2θ) from 5° to 50° in an X-ray XPert Diffractometer (Malvern PANalytical, Malvern, United Kingdom). XRD deconvolution was employed to determine the degree of crystallinity (XC) of the samples. Initially, the deconvolution of the pristine SS and PVA samples was conducted using OriginPro 2016 software. Based on these peaks and those reported in the literature (Table 1), the areas corresponding to the amorphous and crystalline peaks of crosslinked and non-crosslinked SS/PVA biohydrogels were then calculated. Equation (1) was used to compute the XC for samples, where *A_c_* and *A_a_* are the areas of the crystalline and amorphous regions, respectively [43].

(1) $$XC\left(\%\right)=\frac{A_{c}}{A_{c}+A_{a}}*100$$

#### 2.5.2. Fourier-Transform Infrared Spectroscopy (FTIR)

The chemical structures of the crosslinked and non-crosslinked SS/PVA hydrogels were analyzed using FTIR with an attenuated total reflectance module (ATR) on a Nicolet 6700 Series brand spectrometer (Thermo Electron Corporation, Beverly, MA, USA). A total of 64 scans were performed at a resolution of 4 cm^−1^ and a wavelength range of 4000–400 cm^−1^. This was conducted in order to study their secondary structures, as reported by other authors [48,49,50]. The sensitivity of the amide I vibration to the secondary structure makes it possible to study protein folding, unfolding, and aggregation [51]. To achieve this, deconvolution with Gaussian curves in the amide I region (1600–1700 cm^−1^) was evaluated using OMNIC^®^ V9.5 software. Briefly, deconvolution was performed using a gamma function of 20 and a noise reduction factor of 0.3. After extraction of a straight baseline in the deconvoluted amide I region, a second derivative was applied to identify the position of the maximum band. Spectra were then fitted using seven Gaussian curves, and the relative content of each secondary structure was calculated as the ratio between the area of the associated peaks ($A_{m}$) and the total area ($A_{T}$). All measurements were performed in triplicate. Table 2 lists the wavenumber ranges assigned to the vibrational bands of the secondary structures of amide I, indicating the type of structure (crystalline or amorphous).

#### 2.5.3. Scanning Electron Microscopy (SEM)

The secondary electrons HV-SEM technique (JSM-6490LV, JEOL, Tokyo, Japan) was used to observe the morphology of SS/PVA hydrogels at 5 kV. Prior to analysis, the hydrogels were carefully cut in a horizontal plane using a razor blade. Samples were covered with a thin layer of gold using Desk IV equipment (DENTONVACUUM, Moorestown, NJ, USA), until a thickness of approx. 10 nm was obtained. Images were collected at 100× magnification and analyzed using the open-source software ImageJ V1.53. In total, 100 pore widths were measured for each sample. In addition, the chemical compositions of the cross sections of the samples were evaluated using SEM with energy-dispersive X-ray spectroscopy (SEM/EDX).

#### 2.5.4. Thermogravimetric Analysis (TGA)

The thermal behavior of the samples was determined using a thermogravimetric analyzer (TGA-Q500, TA Instruments, New Castle, DE, USA). A total of 10 mg of each sample was processed under an inert atmosphere of N_2_ to avoid oxidative processes, at a rate of 50 mL/min, with a temperature range of 30 to 800 °C using a 10 °C/min ramp. Using the results obtained from the TGA, the first mass derivative (DTG) was calculated with respect to time to obtain the decomposition profile and characteristic temperatures.

#### 2.5.5. Water Absorption Capacity

The water absorption analysis was adapted from Mandal, Priya and Kundu [52]. Briefly, the dry mass of the hydrogels was recorded prior to their immersion in distilled water for 24 h. During the treatment, the samples were held on a metal grill to facilitate handling. After this time interval, the hydrogels were carefully extracted from the solution, the excess water was removed with a cotton towel, and the mass of the samples was immediately recorded. The percentage of water absorption capacity was calculated using Equation (2).

(2) $$Water\;absorption\;capacity\left(\%\right)=\frac{M_{w}-M_{0}}{M_{0}}*100$$

where *M_w_* (g) and *M*_0_ (g) are the mass of the samples in wet and dry initial states, respectively.

After the water absorption test, the wet samples were dried at 60 °C in a forced-convection oven (BOV-V30F, Biobase, Jinan, China) for 24 h and weighed (*M_d_*) to evaluate the change in their mass. The mass loss was calculated using Equation (3). All assays were performed in triplicate (*n* = 3).

(3) $$Mass\;loss\;(\%)=\frac{M_{0}-M_{d}}{M_{o}}*100$$

#### 2.5.6. Silk Sericin Release from the Hydrogels

To evaluate the release of SS from the crosslinked and non-crosslinked hydrogels, the hydrogels were immersed in 9 mL of 1× PBS solution (pH 7.4) in a controlled environment at a temperature of 37 °C. After 24 h, 1 mL solution samples were taken from each test [53]. The amount of SS released from materials in the PBS solution was determined by the biuret method. This method is commonly used to detect and quantify the presence of proteins in a solution by reacting the peptide bonds of the protein with a copper-containing reagent in an alkaline environment, resulting in the formation of a purple-colored complex [54]. Initially, a calibration curve was performed using a standard protein solution with a concentration of 2% (*w*/*v*) [33]. The solutions were left to rest for 30 min at room temperature to ensure color development, which was read using a UV-VIS spectrometer (DR 2700, Hach, Loveland, CO, USA). The absorbance reading of 1 mL of PBS added to 10 mL of PBS was set as a blank at 510 cm^−1,^ and it was used to adjust the 0.0 absorbance level. All experiments were performed in triplicate. The percentage of SS released from the hydrogels was calculated based on the initial amount of SS in the materials (see Equation (4)).

(4) $$Cumulative\;released\;SS\;(\%)=\frac{M_{p}}{M_{m}}*100$$

where *M_p_* is the amount (mg) of determined protein with the biuret method and *M_m_* is the initial amount (mg) of protein present in the material.

#### 2.5.7. Compressive Strength

Compression tests were performed on the C, T, V.E, and V.A biohydrogels using a universal testing machine (Instron 5582, Norwood, MA, USA) at a constant compression rate of 1 mm/min [55]. The compression endpoint was set at 80% of the initial hydrogel thickness. This test was performed in triplicate, and the results are reported as the average with standard deviations.

#### 2.5.8. Statistical Analysis

To probe the differences between process conditions applied in the secondary structure analysis, water absorption capacity, mass loss, compressive strength, and protein release, a one-way ANOVA with multiple comparisons was used (LSD Fisher test with a 5% confidence level) using Origin (2016 version) software. A minimum of three non-dependent tests were performed.

## 3. Results and Discussion

### 3.1. Impact of Physical Crosslinking on the Structure of Biohydrogels

**Figure 2:**
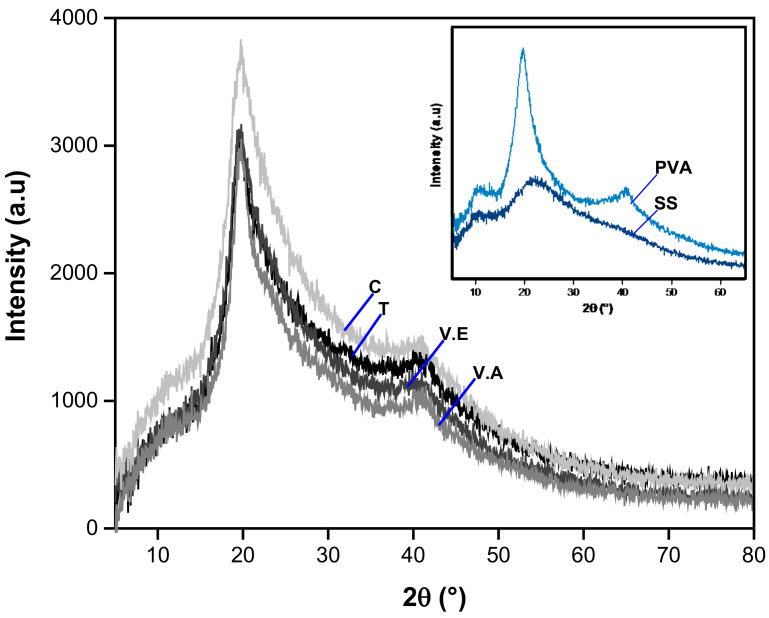
XRD diffraction patterns for pristine SS and PVA hydrogels, crosslinked using annealing treatment (T), exposure to an absolute ethanol vapor atmosphere (V.E), water vapor annealing (V.A), non-crosslinked (C) SS/PVA hydrogels.

The XRD patterns of pristine SS and PVA, SS/PVA control, and T, V.E, and V.A crosslinked hydrogels are shown in Figure 2. For the pristine SS hydrogel, one wide peak was observed around 2θ = 22° and another lower peak was observed at 10°. According to recent research, pure sericin materials exhibit a mainly amorphous structure with few β-structures [33,46]; thus, the XRD pattern might not have a defined peak and may present a broad peak. Three typical peaks (2θ around 12.5°, 22.6°, and 40.6°) are observed in the XRD pattern of pristine PVA, corresponding to the plans of PVA crystallites [29,48]. For C hydrogel, high- and low-intensity peaks are observed around 19.9° and 40.6°, respectively, and a flattened lower peak at 10° is also evidenced. Moreover, the peak at 22.6° in the PVA hydrogel is displaced in the C hydrogel (19.9°); this could be due to the influence of the low crystallinity of sericin in the C hydrogel. Similar behavior has been observed in a study of PVA hydrogels blended with collagen hydrolysate and sericin [29]. The authors suggest that higher protein content samples tend to decrease both crystallinity and crystallite size [29].

Regarding the influence of crosslinking processes, the T, V.E, and V.A hydrogels show the same position of the three peaks evidenced in C hydrogel (2θ around 10°, 19.9°, and 40.6°) but are considerably narrower. Consequently, in the XC analysis, it was found that the C samples exhibit an XC of 67.05%, whereas all treated samples exhibit an increase in this parameter, resulting in values of 69.33, 67.46, and 70.43% for T, V.E., and V.A hydrogels, respectively. This last sample showed the highest increase in the XC compared with the control hydrogel. These results indicate that the crystallinity of the polymer blend is significantly affected by the crosslinking method used, possibly increasing the crystalline domains. To confirm this hypothesis, a detailed analysis of both conformations and structures in the hydrogels was performed using Fourier-transform infrared spectroscopy (FTIR) and thermogravimetric analysis (TGA).

**Figure 3:**
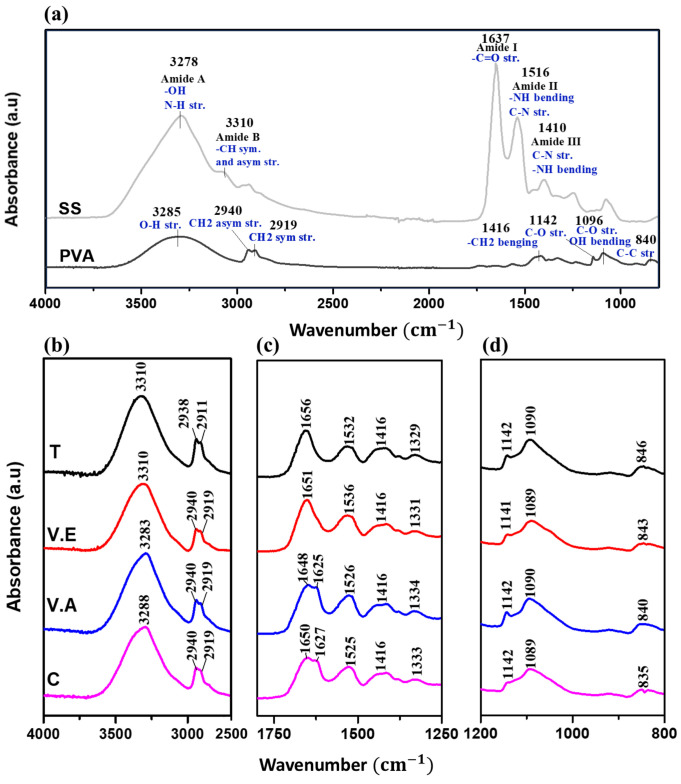
FTIR spectra for (**a**) pristine SS and PVA hydrogels, crosslinked with annealing treatment (T), exposure to an absolute ethanol vapor atmosphere (V.E), water vapor annealing (V.A), and non-crosslinked (C) SS/PVA biohydrogels in three different ranges of wavenumbers (**b**) 4000–2500 cm^−1^, (**c**) 1800–1250 cm^−1^, and (**d**) 1250–800 cm^−1^.

The spectra FTIR obtained for the SS and PVA-based hydrogels are shown in Figure 3. The characteristic vibrations of pristine SS and PVA hydrogels are shown in the spectra of Figure 3a. For crosslinked (T, V.E, and V.A) and control SS/PVA hydrogels, spectra are presented in the ranges of 4000–2500 cm^−1^ (Figure 3b), 1800–1250 cm^−1^ (Figure 3c), and 1200–800 cm^−1^ (Figure 3d).

In Figure 3a, SS hydrogel produces multiple characteristic infrared absorption bands, including amide A and B (3000–3500 cm^−1^), with a higher intensity peak to 3278 cm^−1^ assigned to -OH stretching and the strong hydrogen bond in the β-sheet structures of the SS hydrogels, and N-H stretching vibrations [48,56,57,58]. This peak is shifted in the T, V.E, V.A, and C hydrogels (Figure 3b), evidencing that the blending and physical treatment promote hydrogen bonding interactions [18]. Among these, the T and V.E hydrogels exhibit the most significant peak displacement (3310 cm^−1^) compared to the V.A and C hydrogels. This result suggests a stronger interaction with water, attributed to the increases resonance frequency associated with hydrogen bond interactions [17].

PVA presents a peak at 3285 cm^−1^ due to O-H stretching, and characteristic vibrations at 2940 and 2919 cm^−1^ attributable to asymmetric and symmetric stretching of CH_2_, respectively [59]. The bands assigned to asymmetric and symmetric stretching of CH_2_ for the T hydrogel show a shift in wavelength to the right (2938 and 2911 cm^−1^) while C, V.E, and V.A hydrogels maintain the same vibration as pristine PVA hydrogel.

In protein molecules, the amide bonds contribute to the conformational changes in the polypeptide backbone [29]. In Figure 3a, amide I (1700–1600 cm^−1^) and amide II (1600–1500 cm^−1^) are characteristic of the sericin FTIR spectrum, and specific vibrations are observed at 1637 cm^−1^ (C=O stretching vibrations of peptide linkages) and 1516 cm^−1^ (N-H bending and C-N stretching vibrations), evidencing that secondary amorphous structures (random coil) are predominant in pristine SS hydrogel [60]. Figure 3c shows that amide I band of T and V.E hydrogels at 1656 and 1651 cm^−1^, respectively, are shifted to higher wavenumbers when compared to the SS hydrogel. This same behavior was observed for C (1650 cm^−1^) and V.A (1648 cm^−1^) hydrogels, in which an additional shoulder was evident at 1627 cm^−1^ and 1625 cm^−1^, respectively; this result suggests the presence of more ordered structures in the conformation of the protein. The band associated with amide II also appears at a higher wavelength for all composite hydrogels (see Figure 3c). According to different authors, these results are due to the formation of hydrogen bonds between the protein and PVA; the hydrogen bonding increases the frequency of bending vibrations since it produces an additional restoring force [17,51]. For PVA, characteristic absorption peaks are at 1564 cm^−1^ (due to water absorption), 1416 cm^−1^ (CH_2_ bending), and 1327 cm^−1^ (δ (OH), rocking with -CH wagging) [60,61,62]. Similarly, the sericin-based hydrogels with and without crosslinking present the same band at 1416 cm^−1^, while the peak at 1327 cm^−1^ for SS hydrogel presents a shift to a higher wavelength in all SS/PVA hydrogels.

Moreover, PVA shows distinct intense peaks: at 1142 cm^−1^, which represents the crystalline sequence of the polymer (shoulder stretching of C-O); at 1096 cm^−1^, which represents the amorphous sequence (stretching of C-O and bending of OH); and at 835 cm^−1^ associated with C-C stretching [58,60,61]. Notably, the peak at 1142 cm^−1^ is present in the C, T, V.E, and V.A hydrogels (see Figure 3d), where the T and VA hydrogels present a higher intensity in this peak compared to the C hydrogel, indicating the increase in the crystalline rearrangement of PVA. In addition, the peaks mentioned above at 1096 and 835 cm^−1^ show a shift to a lower and higher wavelength, respectively, in all physically crosslinked hydrogels.

**Figure 4:**
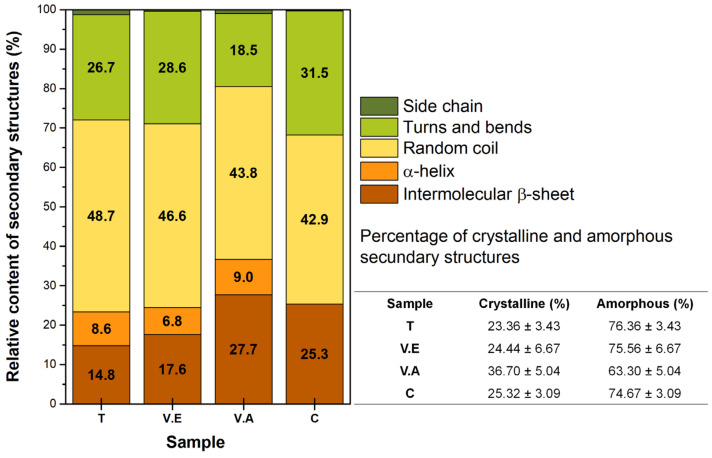
The relative content of secondary structures for hydrogel crosslinking with annealing treatment (T), exposure to an absolute ethanol vapor atmosphere (V.E), water vapor annealing (V.A), and non-crosslinking (C), and a summary of crystalline and amorphous structures.

According to these results, it is necessary to carry out an analysis of the rearrangement that produces crosslinking processes on the secondary structure of sericin in hydrogels. A quantitative study was performed using deconvolution of the amide I region (1700–1600 cm^−1^). The relative content of secondary structures for crosslinked (T, V.E, and V.A) and non-crosslinked (C) hydrogels and a summary of the crystalline and amorphous structures are shown in Figure 4.

The C hydrogel presents a high content of amorphous structures (74.67 ± 3.09%), similar to T and V.E. hydrogels, where random spiral structures predominate, indicating that the SS chains distort the crystalline phase of PVA [29]. In the V.A hydrogels, a rearrangement process occurs for both polymers, obtaining a higher content of intermolecular parallel β-sheet structures due to the water vapor molecules that can transfer thermal energy into the protein-bound water system. The water molecules interrupt the intermolecular cohesive forces between the protein chains and reduce the steric effect for movement and reorientation, improving the mobility of the amorphous domains, and favoring crystallization with PVA [31,62,63,64].

**Figure 5:**
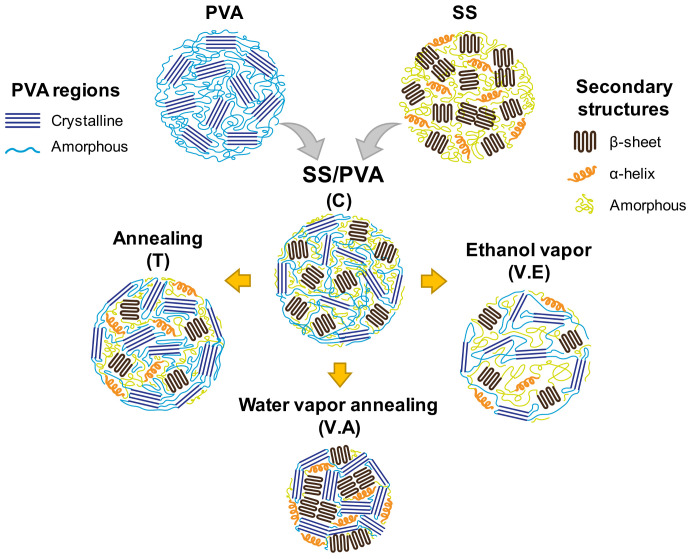
Illustrations of the structural conformation of SS/PVA hydrogels crosslinking with annealing treatment (T), exposure to an absolute ethanol vapor atmosphere (V.E), water vapor annealing (V.A), and non-crosslinked (C).

V.E hydrogels present an expansion of the amorphous region of SS with a predominant content of random coil structures (~46.6%). These results are due to the interruption of hydrogen bonds, followed by the penetration of ethanol vapor into the expanded region, generating a hydrophobic environment with a high content of packed amorphous structures [33,65]. This implies that PVA presents greater mobility than sericin, affecting the reorganization capacity of the protein. Finally, the T hydrogel presents the highest content of sericin amorphous structures (76.36 ± 3.43%) owing to the constant temperature. This treatment promotes the alignment and folding of the PVA macromolecular chains, causing PVA crystallization while stabilizing the amorphous structure of SS. According to the above, Figure 5 illustrates the regions and structures of the SS/PVA polymers and biohydrogels.

**Figure 6:**
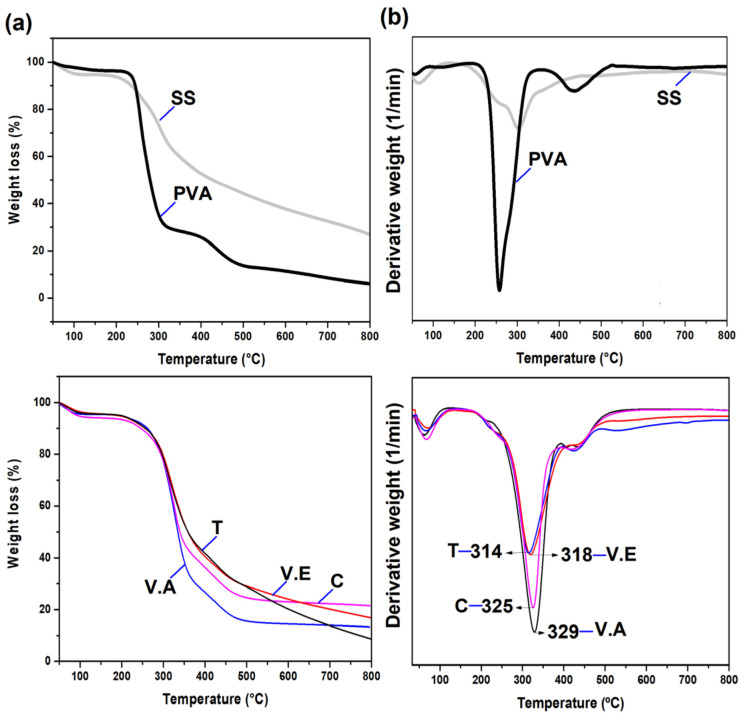
(**a**) TGA and (**b**) DTG curves for pristine SS and PVA hydrogels, and crosslinking with annealing treatment (T), exposure to an absolute ethanol vapor atmosphere (V.E), water vapor annealing (V.A), and non-crosslinked (C) hydrogels.

Thermogravimetric analysis (TGA) and a derivate (DTG) of T, V.E, V.A crosslinked, and C non-crosslinked hydrogels are presented in Figure 6. All the SS/PVA-based hydrogels present a first stage of decomposition before 200 °C, with a weight loss of 3–6% associated with free and bound water evaporation [33,66]. The second stage of decomposition (225 to 470 °C) of the SS/PVA-based hydrogels is related to the elimination of volatile compounds, degradation of amino acid residue side chains of sericin, and decomposition of PVA side chains [2,17].

According to the DTG curves, a first shoulder is observed at approximately 257, 246, 246, 246, and 225 °C for C, T, V.E, and V.A hydrogels, respectively. Although for this first shoulder there are no reports associating specific decomposition mechanisms, it is believed that it may be related to the cleavage of the peptide bonds of the lower molecular weight protein [56]. This may also be related to the degradation and mobility of the PVA chains since pristine PVA has a maximum decomposition temperature (T_max_) of 257 °C and occurs almost in a single step.

On the other hand, the T hydrogel shows the lowest T_max_ at 314 °C, followed by V.E, C, and V.A hydrogels (318, 325, and 329 °C, respectively). The difference between the T_max_ of hydrogels is related to the degree of crosslinking between the SS and PVA chains, where high crosslinking restricts the movement of polymer chains, and more thermal energy is required to cause changes, increasing thermal stability. Additionally, it is believed that the degree of SS crystallization in the sample and thermal stability are closely related [30]. A final peak is evident around 419, 427, 435, and 407 °C for the C, T, V.E, and V.A hydrogels, respectively, which is characteristic of the final phase of thermal decomposition of PVA [67]. This confirms the results of the FTIR analysis, especially the physical crosslinking of the polymers in the blend and the strong affinity between SS and PVA.

### 3.2. Morphological Analysis

**Figure 7:**
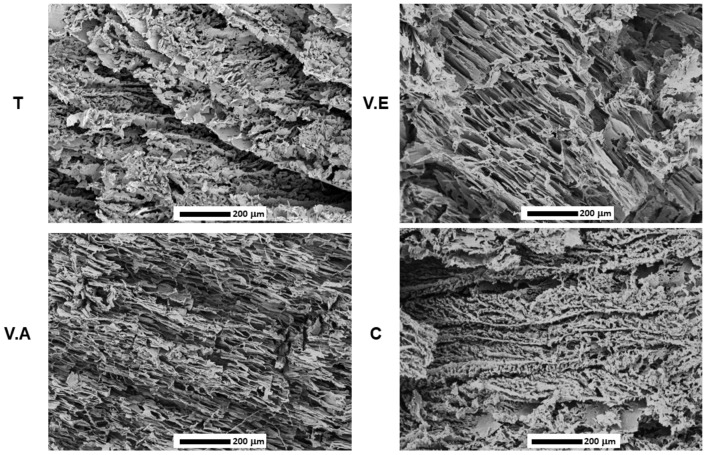
Morphological observation by SEM of cross-section images of SS/PVA hydrogels crosslinking with annealing treatment (T), exposure to an absolute ethanol vapor atmosphere (V.E), water vapor annealing (V.A), and non-crosslinked (C) hydrogels.

The morphologies of the cross-section crosslinked (T, V.E, and V.A) and non-crosslinked (C) hydrogels are shown in Figure 7. All the SS/PVA-based hydrogels present a porous microstructure associated with the polymer mixture and the manufacturing method, and different pore configurations due to the influence of the crosslinking process were evident. The C hydrogel exhibits small cavities between the sheets, forming partially interconnected heterogeneous porosities, confirming the hydrogen bonding interactions described in the FTIR analysis [30]. The presence of this type of interaction influences the arrangement of the polymer chains and the formation of void spaces within the hydrogel. For the T hydrogel, the results show that the annealing treatment favored an ordering of the structure with small pore interconnections and an increase in interlayer voids compared to the C hydrogel, which could be due to the movement of the polymer chains mainly of PVA during the crosslinking process at a high temperature [24,35].

Regarding the V.A hydrogel, more elongated porosities with a smaller average pore width (approx. 10.08 ± 6.65 μm) and interlayer contractions were observed. It has previously been shown that water vapor annealing causes compression of the three-dimensional structure (see Figure 1) owing to interlayer contraction, which is caused by reducing the steric space between the polymer chains during the treatment. According to recent studies, the treatment of protein-based materials with water vapor annealing generates sheet-like structures with orientation and a decrease in pore size [62,68]. This is in agreement with the obtained results. Similar outcomes are observed for the V.E hydrogel, which shows a thicker pore wall and elongated porosities (average pore width11.03 ± 8.26 μm). In addition, V.E shows shrinkage between the layers, although to a lesser extent than the V.A hydrogel, which could be explained by the hydrophobic dehydration that occurs due to molecular interactions between the protein chains and the polar solvent [33,69]. The morphological analysis and the value of the mean pore size for the crosslinked and uncross-linked hydrogels are summarized in Table 3.

**Figure 8:**
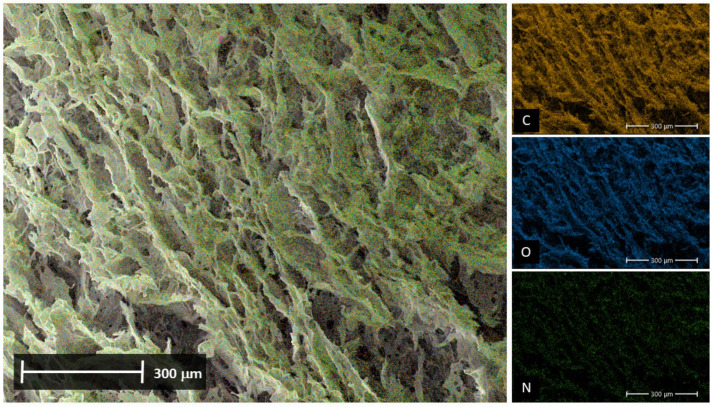
EDX analysis of non-crosslinking control biohydrogels. The distribution of elements—carbon, oxygen, and nitrogen—in the SS and PVA polymers within the composite C-hydrogel is presented.

In addition to morphological analysis, a cross-sectional area of the SS and PVA composite samples was selected and mapped to determine their elemental composition using EDX. Typical elements for each composite are observed in both treated and control samples. Carbon (C), oxygen (O), and hydrogen (H), which are elements common to both composites, are detected, and nitrogen (N), an element unique to the SS protein, is also homogeneously evident (see Figure 8). These results suggest that the formation of the materials results in a uniform polymer blend.

### 3.3. Performance Analysis

#### 3.3.1. Compressive Strength

**Figure 9:**
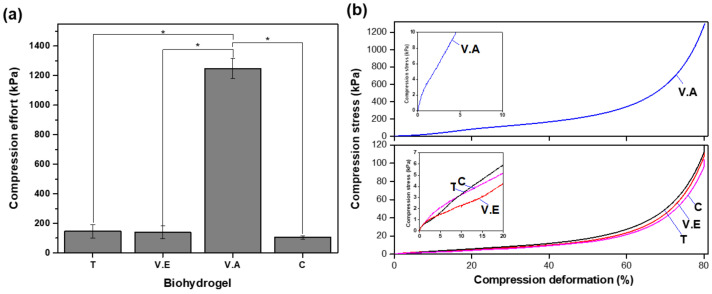
Mechanical integrity (**a**) compressive stress at 80% strain and (**b**) curves of T, V.E, V.A crosslinked, and C biohydrogels. The bars represent the standard deviation (*n* = 3) and the asterisks refer to the statistically significant difference. * *p* < 0.05 significant differences.

Figure 9 shows the compression effort at 80% strain (Figure 9a) and compression stress–strain curves (Figure 9b) for the crosslinked and non-crosslinked biohydrogels. Generally, biomaterials tend to exhibit consistently increasing deformation with increasing compression. The V.A hydrogel presented the highest compressive effort of (1248 ± 6.81) kPa, at 80% of its deformation, compared to the T, V.E, and C hydrogels.

Sericin is known to decrease the mechanical properties of materials, whereas PVA provides greater mechanical strength, and its affinity provides structural improvement, especially when physically crosslinked [57,67,70]. The stiffness of biomaterials is indicated by the slope of the initial portion of the stress–strain curve in the elastic zone; the steeper the slope, the greater the stiffness. In this case, V.A shows a steep curve in the initial portion of the curve, with minimal plastic deformation (Figure 9b). This sample exhibits a significant increase in stress with a small amount of deformation, which is characteristic of a very stiff material. In contrast, the T, C, and V.E hydrogels present lower stiffness, with V.E being the least stiff and most ductile, as indicated by their relatively gentle slopes in the initial portions of the curves. The T and C hydrogels exhibit clear plastic zones starting after 2–3% deformation, demonstrating a more gradual increase in stress and indicating permanent deformation compared to the V.A hydrogel. The V.A hydrogel stands out among the other crosslinking methods evaluated because of the formation of a compact and porous structure in which the crystallization and binding sites of both polymers predominate.

#### 3.3.2. Water Absorption Capacity and Mass Loss

**Figure 10:**
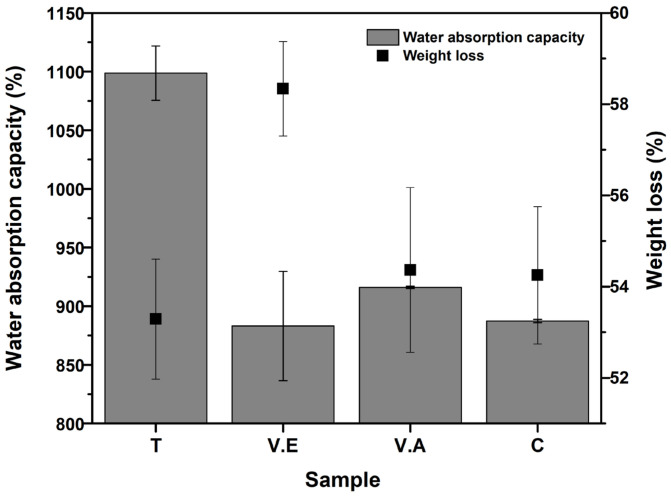
Water absorption after 24 h of immersion in water and mass loss after drying for SS/PVA hydrogels crosslinking with annealing treatment (T), exposure to an absolute ethanol vapor atmosphere (V.E), water vapor annealing (V.A), and non-crosslinked (C) hydrogels. The bars represent the standard deviation (*n* = 3).

The percentage of water absorption after 24 h of immersion in water and mass loss after drying at 60 °C for T, V.E, V.A crosslinked and C non-crosslinked hydrogels are shown in Figure 10. All biohydrogels rapidly absorbed water upon immersion due to the hydrophilic nature of polymers and the large surface area of the samples [53,71]. V.E, V.A, and C hydrogels exhibit absorptions of (883 ± 4.65)%, (916 ± 1.02)%, and (887 ± 1.43)%, respectively, without statistical differences between them. Due to increased crystallinity and reduced pore size, crosslinking methods commonly used in protein-based hydrogels reduce water absorption capacity [68,71,72,73]. However, as mentioned above, the annealing treatment created a morphology composed of large interlayer voids (see SEM analysis), allowing greater water absorption (1098 ± 2.31)%. The physical properties of the resulting materials, such as water absorption, are mainly influenced by their morphological features and play an important role in their potential use as absorbent materials. In wound dressing, the water absorption capacity promotes the removal of excess exudate, promotes oxygen permeation, and maintains adequate moisture at the wound site [4,74].

On the other hand, the V.E. hydrogel shows the highest mass loss (58.33 ± 1.03%) compared to T, V.A., and C hydrogels. This result is due to the fact that ethanol vapor treatment weakens the crystalline domains and expands the amorphous region of the protein, which can provide mass loss associated with the release of sericin from the material (see Section 3.3.3).

There was no statistically significant difference in mass loss between T, VA, and C hydrogels. In addition, it is possible that the mass loss observed in all hydrogels is related to the solubility of PVA owing to the content of free hydroxyl groups (-OH) in its structure [67]. It has been shown that physical crosslinking improves the water stability of protein-based hydrogels [34,35,68,75]. This assessment differs from the results obtained in our study, mainly due to the way in which the treatment interacts with the SS and PVA polymers individually and together.

#### 3.3.3. Silk Sericin Release from the Hydrogels

**Figure 11:**
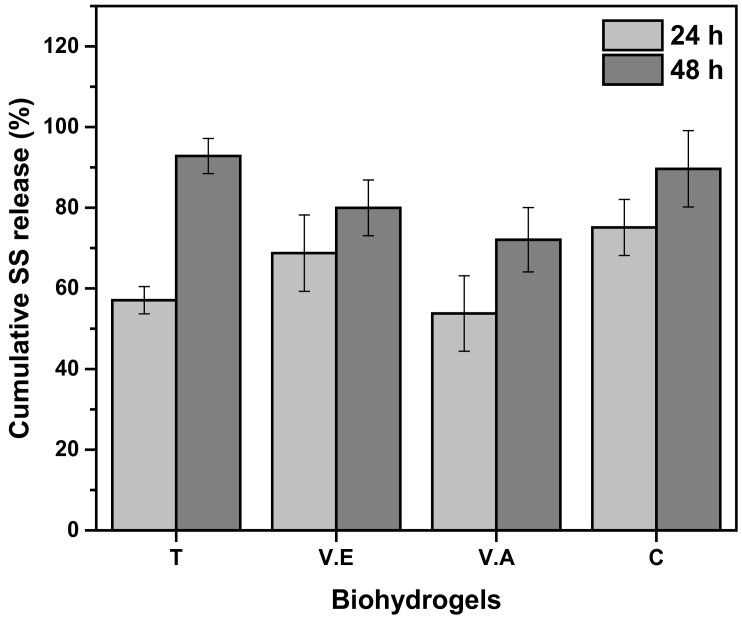
Percentage of sericin released with respect to the initial proportion of protein in the SS/PVA biohydrogels crosslinking with annealing treatment (T), exposure to an absolute ethanol vapor atmosphere (V.E), water vapor annealing (V.A) and non-crosslinked (C) hydrogels.

Several researchers have demonstrated the use of sericin as a bioactive compound due to its attractive physicochemical properties [76]. In addition, sericin has been explored as a drug delivery or encapsulation material in various biomedical applications owing to the presence of strong polar side chains such as hydroxyl, carboxyl, and amino groups, as well as its high hydrophilicity, which improves the properties of the polymer matrix [77]. Figure 11 shows the sericin release profile of crosslinked and non-crosslinked SS/PVA-based hydrogels after 24 and 48 h of immersion in PBS.

The results show a greater release of sericin from C (75.09 ± 6.95%) at 24 h, followed by the V.E hydrogel (68.74 ± 9.06%), without statistical difference (*p* value > 0.05). The T and V.A hydrogels showed a lower release percentage at 24 h, (57.05 ± 3.39) and (53.77 ± 9.34)%, respectively. The following order, from the highest to the lowest percentage of release, was found for the hydrogels after 24 h of immersion: C > V.E > V.A > T. On the other hand, the T hydrogel was found to have the highest percentage of sericin released (92.81 ± 4.36%) along with the C hydrogel (90%) after 48 h of immersion, compared to the V.A and V.E hydrogels. This behavior is ascribed to the sericin in the T hydrogel is trapped between the crystalline chains of PVA, but its structure is amorphous (76.56% of random coil structures), resulting in increased release when most exposed to immersion. While for the V.A hydrogel, sericin presents a higher content of crystalline structures (intermolecular b-sheet), which provides a lower release of the protein in PBS. In the case of the V.E hydrogel, this treatment affects the PVA structure, generating weak crystalline domains and an amorphous state of sericin. As a result, there was no difference in the release profile of the V.E hydrogel compared with that of the C hydrogel.

## 4. Conclusions

In this study, three environmentally friendly crosslinking methods—annealing treatment (T), ethanol vapor (V.E), and water vapor annealing (V.A)—were evaluated to improve the physicochemical properties and performance of biohydrogels composed of a 50/50 SS/PVA mixture.

The presence of both silk sericin (SS) and poly (vinyl alcohol) (PVA) in the hydrogels and the structural changes generated by the crosslinking processes were confirmed by FTIR. The T hydrogel showed that PVA crystallization predominated and stabilized the amorphous structure of SS. In the V.A hydrogel, a rearrangement process occurred for both polymers, resulting in a higher content of intermolecular parallel β-sheet structures. The V.E hydrogel presented an expansion of the amorphous region of the SS owing to the interruption of the hydrogen bonds, followed by the penetration of ethanol vapor in the expanded region. Finally, the control hydrogel formed hydrogen bonds with SS, which distorted the crystalline phase of PVA. TGA was dominated by the constraints of the crosslinking chains, where it was evident that the V.A hydrogel presented a higher decomposition temperature, followed by the non-crosslinking (C), V.E, and T hydrogels.

The results of morphology revealed that annealing treatment favors the formation of large interlayer voids, with an interconnection of pores, which increased the water absorption capacity during 24 h of immersion, compared to V.A, C, and V.E hydrogels, as confirmed by studies on water absorption capacity. The V.E hydrogel presented a higher mass loss in water after 24 h of immersion, which was mainly due to the high content of amorphous structures in the hydrogel. The release of sericin from the tested hydrogels in a PBS solution at 24 and 48 h revealed a slower release percentage in the T hydrogel compared to V.A, V.E, and C hydrogels. However, all biohydrogels showed complete release of SS after 48 h of immersion. It is important to determine in future research how the loss of SS in the hydration processes affects the mechanical properties of the material and guides its application to those that can take advantage of the bioactive properties of this protein in a short period. Compression strength demonstrated that the V.A hydrogel presented the highest compressive strength at 80% of its deformation compared with T, V.E, and C hydrogels, which did not present statistically significant differences.

It was demonstrated that V.A and T treatment stand out because they improve the absorption and stability in water, with a slower release of SS. These results suggest that physical crosslinking induces conformational transitions and morphological changes. Consequently, the treated SS/PVA hydrogels exhibit characteristics that allow these materials to be used as biomaterials for wound healing applications, and the crosslinking conditions must be considered according to the requirements or specific properties of the biomaterial.

## Figures and Tables

**Table 1:** X-ray diffraction peaks of SS and PVA [29,33,44,45,46,47].

Region	Diffraction Peak (2θ)	Polymer
Crystalline	12.0	SS and PVA (overlapping)
	19.7	PVA
	22.0	SS and PVA (overlapping)
	40.6	PVA
Amorphous	31.0	PVA
	44.0	SS

**Table 2:** Assignment of amide I position to secondary structures and crystalline or amorphous structures.

Assignment	Wavenumber Ranges (cm^−1^)	Type of Structure
Side chain	1590–1605	Amorphous
Intermolecular β-sheet	1610–1625 and 1697–1700	Crystalline
Random coil	1636–1645	Amorphous
α-helix	1658–1664	Crystalline
Turns and bends	1666–1695	Amorphous

Adapted from [49].

**Table 3:** Summary of morphological analysis of SS/PVA-based hydrogels using annealing treatment (T), exposure to an absolute ethanol vapor atmosphere (V.E), water vapor annealing (V.A), and non-crosslinked (C) hydrogels.

Type of Hydrogel	Average Pore Diameter (mm)	Morphology
T	Does not apply	Heterogeneous pores—interlayer voids
V.E	11.03 ± 8.26	Elongated pores—interlayer contraction
V.A	10.08 ± 6.65	Elongated pores—interlayer contraction
C	Does not apply	Heterogeneous pores—small cavities

## Data Availability

The data will be made available upon request.

## Abbreviations

SS, silk sericin; T, annealing treatment; V.E, exposure to an absolute ethanol vapor atmosphere; V.A, water vapor annealing; PVA, polyvinyl alcohol; FTIR, Fourier-transform infrared spectroscopy; TGA, thermogravimetric analysis; DTG, first mass derivative of TGA curve; SEM, scanning electron microscopy; PBS, phosphate-buffered saline; XRD, X-ray diffraction; XC, degree of crystallinity.
